# Chemical Composition and Antibacterial Activity of Essential Oils of* Tagetes minuta* (Asteraceae) against Selected Plant Pathogenic Bacteria

**DOI:** 10.1155/2016/7352509

**Published:** 2016-09-18

**Authors:** Martin Muthee Gakuubi, John M. Wagacha, Saifuddin F. Dossaji, Wycliffe Wanzala

**Affiliations:** ^1^School of Biological Sciences, University of Nairobi, P.O. Box 30197-00100, Nairobi, Kenya; ^2^Department of Biology, Faculty of Science, Mwenge Catholic University, P.O. Box 1226, Moshi, Tanzania; ^3^Department of Biological Sciences, School of Science and Information Sciences, Maasai Mara University, P.O. Box 861-20500, Narok, Kenya

## Abstract

The objective of this study was to determine the chemical composition and antibacterial activity of essential oils (EOs) of* Tagetes minuta* against three phytopathogenic bacteria* Pseudomonas savastanoi* pv.* phaseolicola*,* Xanthomonas axonopodis* pv.* phaseoli*, and* Xanthomonas axonopodis *pv.* manihotis*. The essential oils were extracted using steam distillation method in a modified Clevenger-type apparatus while antibacterial activity of the EOs was evaluated by disc diffusion method. Gas chromatography coupled to mass spectrometry (GC/MS) was used for analysis of the chemical profile of the EOs. Twenty compounds corresponding to 96% of the total essential oils were identified with 70% and 30% of the identified components being monoterpenes and sesquiterpenes, respectively. The essential oils of* T. minuta* revealed promising antibacterial activities against the test pathogens with* Pseudomonas savastanoi* pv.* phaseolicola* being the most susceptible with mean inhibition zone diameters of 41.83 and 44.83 mm after 24 and 48 hours, respectively. The minimum inhibitory concentrations and minimum bactericidal concentrations of the EOs on the test bacteria were in the ranges of 24–48 mg/mL and 95–190 mg/mL, respectively. These findings provide a scientific basis for the use of* T. minuta* essential oils as a botanical pesticide for management of phytopathogenic bacteria.

## 1. Introduction

Advancement in agricultural technology coupled with improvement in pest and disease management has greatly enhanced global food productivity in the last few decades. However, despite this, agricultural losses occasioned by plant pathogens have remained an important constraint to the attainment of food security in many parts of the world. The combined preharvest and postharvest crop losses due to plant pathogens are estimated at 10–16% of global production [1, 2]. While the threat of plant pathogenic bacteria to food security is less compared to that posed by phytopathogenic fungi, the economic impact of bacterial plant diseases cannot be underestimated [3].

Although the number of bacterial species has been estimated to vary greatly from tens of thousands to billions, only about 100 of these are known to be plant pathogens [3, 4]. Nevertheless, phytopathogenic bacteria are responsible for huge losses in agriculture because most plants of agricultural importance are susceptible to at least one bacterial disease, and, for some crops, bacterial diseases are mostly the primary cause of yield losses [5]. Many plant pathogenic bacteria display enormous diversity in their life-history strategies and host range and host-pathogen interactions which make their impact on crop loss significant [6]. A single bacterial species can be responsible for hundreds of different plant diseases. For example, about 350 different plant diseases are caused by species within the genus* Xanthomonas* [7]. The most significant Gram-negative genera of bacterial plant pathogens are* Agrobacterium, Erwinia, Pseudomonas,* and* Xanthomonas.* All members of the four genera are single-celled, nonspore forming rods with the size of approximately 2.0 × 0.7 *μ*m. Gram-positive phytopathogenic bacteria on the other hand are mostly represented by members of four genera, namely,* Arthrobacter*,* Clavibacter*,* Curtobacterium,* and* Rhodococcus,* all of which are aerobic [8].

Control of plant bacterial diseases continues to be a major challenge due to several factors such as limited availability of bactericides [9] and development of microbial resistance to presently available chemical pesticides [10]. Furthermore, copper-based pesticides that are commonly used in management of many bacterial plant diseases have in recent years gained low public acceptance because of their toxicity which makes them a great health risk to humans, animals, and the environment [9, 11]. Therefore, a search for, and development of, novel and environmentally friendly alternatives to synthetic bactericides for management of plant pathogenic bacteria is continuing and is of utmost importance. Currently, in addition to other natural sources, plants serve as a starting point in the search for new antimicrobial agents or serve as templates for new, more effective compounds with many plant-based antimicrobial agents having been successfully tested and commercialized [12, 13].

Among the major groups of plant products that have elicited strong interest among researchers in the quest for substitutes to synthetic antimicrobials are products of plants' secondary metabolism such as essential oils [14, 15]. Essential oils (EOs) also known as volatile oils or ethereal oils are aromatic oily liquids obtained from various aromatic plant parts. They are not produced by all plants but, rather, their occurrence is restricted to over 2000 plant varieties from about 60 families. Plant families that are particularly rich in essential oils include Asteraceae, Myrtaceae, Pinaceae, Zingiberaceae, Umbelliferae, Lamiaceae, Apiaceae, Rutaceae, and Poaceae [16]. Essential oils are a complex mixture of mainly terpenes particularly monoterpenes and sesquiterpenes and their oxygenated derivatives such as alcohols, aldehydes, esters, ethers, ketones, phenols, and oxides [10, 15]. It is estimated that more than 1000 monoterpene and 3000 sesquiterpene structures are found in plant essential oils [17]. Essential oils are formed in the protoplasm of secretory cells and are isolated from whole plant or plant parts such as flowers, buds, seeds, leaves, twigs, bark, fruits, and roots by different methods such as steam distillation [16].

In recent years, a wide range of plant essential oils and their constituents have been investigated for their antibacterial properties against an array of plant pathogenic bacteria. For instance, Vasinauskiene et al. [13] found varying levels of antibacterial activity of seven essential oils against isolates of phytopathogenic bacteria:* Erwinia carotovora* subsp*. carotovora*,* Xanthomonas vesicatoria*,* Pseudomonas marginalis* pv.* marginalis*,* P*.* syringae* pv.* syringae*,* P. syringae* pv.* syringae*,* P*.* syringae* pv.* tomato*, and* Bacillus* sp. In another study, essential oils of* Origanum acutidens* (Hand.-Mazz.) Ietsw.,* Origanum rotundifolium* Boiss., and* Origanum vulgare* L. were found to be highly effective against 25 plant pathogenic bacterial strains producing inhibition zone diameters between 8 and 48 mm [18]. The antibacterial activity of the three EOs was attributed to the presence of phenolic components such as carvacrol and thymol. Another study investigated the antimicrobial activities of essential oils from eighteen plants against* Agrobacterium tumefaciens* and* Erwinia carotovora* var.* carotovora* [10]. Remarkable antibacterial activity of the tested essential oils was reported with* E. carotovora* var.* carotovora* being more susceptible to the oils than* A. tumefaciens.* The objective of the current study was to determine the chemical composition of steam-distilled essential oils of* T. minuta* and the antibacterial activity of the oils against three phytopathogenic bacteria:* Pseudomonas savastanoi* pv*. phaseolicola*,* Xanthomonas axonopodis* pv*. phaseoli,* and* Xanthomonas axonopodis *pv.* manihotis*.

## 2. Materials and Methods

### 2.1. Collection of Plant Materials and Extraction of Essential Oils

Aerial parts (leaves, flowers, and stems) of* T. minuta* were collected at the flowering stage from Maseno area (0°0′21.43′′S, 34°36′6.23′′E, and 1524 MASL) Kisumu County, Kenya. Essential oils were extracted using the steam distillation method in a modified Clevenger-type apparatus [19]. Plant materials were cut into small pieces (≈10 cm long) and weighed and approximately 4 kg was loaded into the distillation tank that formed part of the steam distillation setup. The plant materials were subjected to steam distillation with the collection of oils starting after a heating time of about 40 minutes and continued until no more essential oil was obtained. After the distillation process was complete, the volatile essential oils were removed from the top of the hydrosol and dried over anhydrous sodium sulphate (Na_2_SO_4_). The oils were filtered using Whatman filter paper (number 1), collected into airtight glass vials, and stored at −20°C until the time when they were required for chemical analysis and antibacterial bioassays. A subsample of the collected plant materials was taken to the herbarium at the School of Biological Sciences, University of Nairobi, Kenya, for further identification and authentication by a plant taxonomist. A voucher specimen (MMG2015/01) was deposited at the University of Nairobi herbarium.

### 2.2. Plant Pathogenic Bacterial Strains

Three economically important bacterial plant pathogens used as test pathogens in the study were retrieved from the culture collection center at the Plant Pathology Laboratory, Department of Plant Science and Crop Protection, University of Nairobi.* Pseudomonas savastanoi* pv*. phaseolicola* and* Xanthomonas axonopodis* pv*. phaseoli* had been isolated from common bean (*Phaseolus vulgaris* L.) while* Xanthomonas axonopodis *pv*. manihotis* had been isolated from cassava (*Manihot esculenta* Crantz). Confirmation of the identity of the pathogens was done based on their cultural, morphological, and biochemical characteristics.

### 2.3. Retrieval of Test Pathogens and Preparation of Bacterial Inocula

Stock culture of each of the test bacteria maintained at −20°C was retrieved by subculturing on nutrient agar (NA) plates. Three pure colonies of the same morphological type were selected from NA plates and aseptically transferred into test tubes containing 10 mL of Mueller-Hinton broth (MHB) using a sterile loop. The culture tubes were then incubated at 37°C for 24 hours to obtain fresh cultures. McFarland standard was used as a reference to adjust the turbidity of bacterial suspensions to be within the required range. Exactly 0.5 McFarland equivalent turbidity standard was prepared by mixing 0.05 mL of 1% barium chloride dehydrate with 9.95 mL of 1% sulfuric acid. Bacterial suspensions were prepared in sterile saline solution which was prepared by dissolving 0.85 g of NaCl in 100 mL of distilled water and autoclaving for 15 minutes at 121°C and 15 psi pressure. The turbidity of the bacterial suspensions for bioassays was adjusted to 0.5 McFarland standards, equivalent to 1.5 × 10^8^ CFU/mL.

### 2.4. Assessment of Antibacterial Activity of* Tagetes minuta* Essential Oils

Antibacterial activity of* T. minuta* essential oils was carried out using disc diffusion method also known as Kirby-Bauer antimicrobial susceptibility test as described by Souza et al. [20]. Two hundred microliters of bacteria suspension prepared from an overnight culture was adjusted to an optical density equal to 0.5 McFarland standard and uniformly spread on Petri plates (9 cm in diameter) containing MHA using a sterile L-shaped glass rod. Sterile Whatman filter paper discs (number 1, 6 mm in diameter) were each impregnated with 10 *μ*L of undiluted crude* T. minuta* essential oil in a sterile biological safety cabinet. The discs were then aseptically placed at the center of the inoculated culture plates using sterile forceps. Dimethyl sulphoxide (DMSO) was used as a negative control while Enrich BM® (immunomodulator2-bromo-2-nitropropane-1,3 diol), a broad-spectrum bactericide used in the control of bacterial diseases such as halo blight, bacterial wilt, and bacterial spot, was used as a positive control. The plates were refrigerated at 4°C for 2 hours to allow the essential oils to diffuse into the agar medium and finally incubated upside down at 37°C for 48 hours. The tests were conducted in triplicate and measurement of the inhibition zones was done after 24 and 48 hours.

The sensitivity of individual bacteria to the essential oil was ranked based on the inhibition zone values expressed in millimeters (mm) as follows: not sensitive (−) for total zone diameters of ≤8 mm; sensitive (+) for diameters between 8 and 14 mm; very sensitive (++) for zone diameters between 15 and 19 mm; and extremely sensitive (+++) for zone diameters of ≥20 mm [21, 22]. The bioassays were conducted in a biological safety cabinet and in accordance with the protocols of Clinical and Laboratory Standards Institute (CLSI) formerly National Committee for Clinical Laboratory Standards (NCCLS).

### 2.5. Antibacterial Activity at Different Concentrations of* Tagetes minuta* Essential Oils

The activity of* T. minuta* essential oils at seven concentration levels was assessed using the disc diffusion method following the procedure described by Clara et al. [23] with some modifications. Two hundred microliters of bacterial inocula (approximately 10^8^ CFU/mL) was uniformly spread on Muller-Hinton Agar (MHA) Petri plates. Serial dilutions of* T. minuta* essential oil were prepared with DMSO. The essential oils were diluted to the following serial geometric dilutions: 50%, 25%, 12.5%, 6.25%, 3.13%, 1.56%, and 0.78%. Sterile Whatman filter paper discs (number 1, 6 mm in diameter) were impregnated with 10 *μ*L of different essential oil concentrations and aseptically placed at the center of the inoculated culture plates. The plates were placed in a refrigerator at 4°C for 2 hours to allow the essential oils to diffuse into the agar. The plates were then incubated at 28°C for 48 hours. The tests were conducted in triplicate and in addition to evaluating the activity of different concentrations of essential oil against the test bacteria; this bioassay was also used to estimate the minimum inhibition concentrations (MICs) of the EOs on the pathogens [23].

### 2.6. Assessment of the Minimum Inhibitory Concentrations and Minimum Bactericidal Concentration

Tube dilution method as described by Caburian and Osi [24] with some modifications was used to assess the minimum inhibitory concentrations (MICs) and minimum bactericidal concentrations (MBCs) of the essential oils against the test bacteria. Twelve sterile screw-capped falcon tubes (15 mL) were numbered #1 to #11 and the last one was numbered as #13. One milliliter of Muller-Hinton broth was introduced into tubes #2 to #11. One milliliter of* T. minuta* essential oil was pipetted into tubes #1 and #2, and the two tubes were capped and vortexed for 5 seconds; one milliliter was withdrawn from the contents of tube #2 and transferred to tube #3; after capping the tube and mixing by vortexing the contents, one milliliter from the contents of tube #3 was withdrawn and transferred to tube #4 and the process repeated until 1.0 mL from tube #9 was added to tube #10. Fifty microliters of standardized bacterial inocula (approximately 10^8^ CFU/mL) was then introduced into tubes #1 to #11 and to tube #13. To tube #13, 0.5 mL of the standard bactericide Enrich BM prepared according to the manufacturer's instructions was added. Tween 20 (0.05%) was added to the essential oil prior to its application into the tubes to improve the oil solubility. The tubes were then incubated at 37°C for 48 hours.

After incubation, the tubes were examined for growth by observing for any turbidity. The tube with the lowest concentration (highest dilution) of the essential oil at which no visible growth or turbidity was observed was reported as the minimum inhibitory concentration of the oil on the test bacteria [25]. The tubes were shaken to homogenize the contents and 0.01 mL of the contents of each tube was subcultured by streaking Mueller-Hinton agar plates. The plates were incubated at 37°C for 24 hours and then observed for any growth of colonies. Minimum bactericidal concentration was determined as the highest dilution (lowest concentration) of essential oil at which no growth occurred following the subculturing onto MHA plates [25].

### 2.7. Gas Chromatography-Mass Spectrometry Analyses of* Tagetes minuta* Essential Oils

Gas chromatography (GC) coupled to mass spectrometry (MS) was used to establish the chemical composition of the EOs. Three replicates (each taken from a different extraction batch) of 1 mg of* T. minuta* essential oils were separately weighed and diluted in 1 mL of dichloromethane to make a stock solution. From the stock solution, further dilution was made as follows: 100 *μ*l of the stock was topped up to 1 mL with dichloromethane and analyzed on an HP-7890A (Agilent Technologies, Wilmington, USA) GC connected to an HP 5975 C (Agilent Technologies, Wilmington, USA) MS. The gas chromatography equipment was fitted with HP-5MS capillary column; 30 m × 0.25 mm internal diameter; and 0.25 *μ*m film thickness with 5% phenyl methyl silicone as the stationary phase (J&W Scientific, Folsom, USA). The operating conditions were as follows: carrier gas was helium with a flow rate of 1.2 mL/min, constant flow mode; splitless injection mode; oven temperature (35°C for 5 min to 280°C at 10°C/min for 10.5 min with a run time of 50 min); and injection volume (1 *μ*L). The components of the essential oil were identified on the basis of their retention indices (RI) (determined with reference to a homologous series of normal alkanes C_5_–C_31_) and calculated based on a quasi-linear equation proposed by van Den Dool and Kratz [26] for temperature-programmed retention index. Identification of essential oil components was further verified by comparison of their MS fragmentation patterns with those reported in the mass spectra library database (NIST05a and Adams MS HP, USA). Quantitative determination of the constituents was made using the calibration curve of the dose-peak area of a pure compound (1,8-cineole, 99%, Gillingham, Dorset, England), with the relative amount of each individual component expressed as percentage of the peak area relative to the total peak area.

### 2.8. Data Analyses

Data were analyzed using the PROC ANOVA procedure of GENSTAT version 15 and significant differences among means compared using Fisher's Protected LSD at 5% probability level. The growth inhibitory effects of the essential oils against the test bacteria were expressed as mean ± standard error of the mean inhibition zones diameter (mm). Linear regression analysis was performed to determine correlations between different concentrations of essential oil and their overall antibacterial activity assessed as diameters of the inhibition zone. Standard dose-response curves were obtained by plotting essential oil concentrations (mg/mL) against the mean inhibition zone diameters (mm).

## 3. Results and Discussion

### 3.1. Yield, Physical, and Chemical Characteristics of* Tagetes minuta* Essential Oils

A mean percent yield of 0.0594% w/w of essential oil was obtained from aerial parts of* T. minuta*. The EOs yield obtained was much higher than that obtained from previous studies from the same plant species from other parts of Kenya [27, 28] but lower than that obtained in two studies in Iran [29, 30].* Tagetes minuta* essential oils obtained were less dense and insoluble in water. The EOs were however soluble in ethanol, dimethyl sulphoxide, and dichloromethane at a level of 1 : 1 (v/v). The oils exhibited a pale yellowish-orange colour with a citrus-like and turpentine-like odour. The oils were liquid at room temperature (23 ± 2°C) and maintained this state even in storage at −20°C. However, when mixed with DMSO in all the dilution levels used in the study, the essential oils froze when stored at −20°C. The essential oils had a density of 0.76 g/mL.

### 3.2. Antibacterial Activity of* Tagetes minuta* Essential Oils

**Figure 1:**
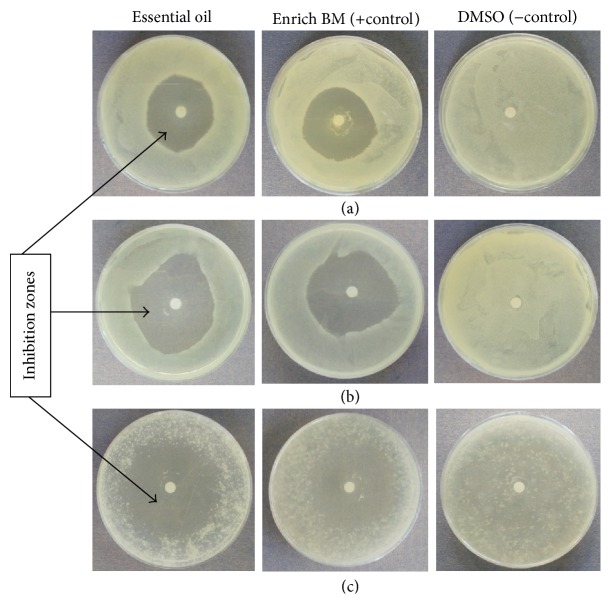
Inhibition zones of* Tagetes minuta* essential oils against* X. axonopodis *pv*. manihotis* (a),* X. axonopodis* pv*. phaseoli* (b), and* P. savastanoi* pv*. phaseolicola* (c) after 48 hours.

*In vitro* studies demonstrated strong antibacterial activity of* Tagetes minuta* essential oils against the three test plant pathogenic bacteria, namely,* Pseudomonas savastanoi* pv*. phaseolicola*,* Xanthomonas axonopodis* pv*. phaseoli,* and* Xanthomonas axonopodis *pv.* manihotis* (Figure 1).

The activity of crude essential oils against the three bacteria isolates was within the extremely sensitive category (diameters of the inhibition zone larger than 20 mm) after 24 and 48 hours of incubation (Table 1). The highest antibacterial activity of crude EOs was observed in* P. savastanoi* pv*. phaseolicola* with mean inhibition zone of 41.8 mm while, for* X. axonopodis* pv*. phaseoli* and* X. axonopodis *pv.* manihotis*, the oils produced inhibition zones of 26.8 mm after 24 hours of incubation. Antibacterial activity of* T. minuta* EOs has been reported in literature with most studies conducted on human pathogenic bacteria [31, 32]. Studies on the antimicrobial activity of essential oils from* T. minuta* and other plant species have generally revealed that Gram-positive bacteria are more susceptible to the effects of EOs in comparison to Gram-negative bacteria [31–33]. The three bacteria used in the current study were all Gram-negative; hence a comparison of this nature was not feasible.

**Figure 2:**
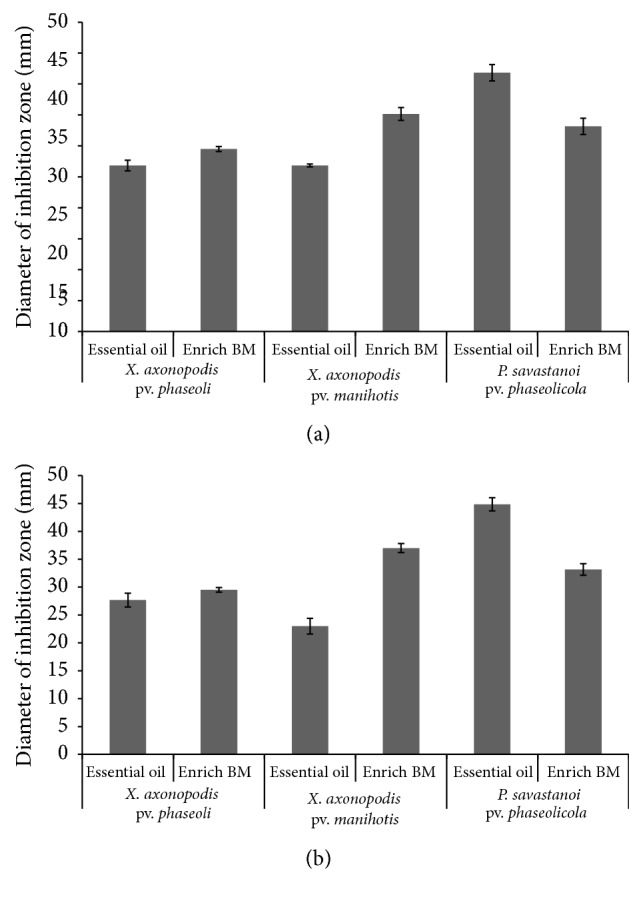
Growth inhibition of the test bacteria by* T. minuta* essential oils and Enrich BM after 24 hours (a) and 48 hours (b). Error bars represent standard error of mean.

The activities of the EOs on* X. axonopodis* pv.* phaseoli* and* P. savastanoi* pv.* phaseolicola* were significantly higher (*p* ≤ 0.05) after 48 hours in comparison to those recorded after 24 hours. However, there was no significant difference (*p* ≥ 0.05) in the antibacterial activity of Enrich BM on* X. axonopodis* pv.* phaseoli* and* P. savastanoi* pv.* phaseolicola* in the two incubation regimes. Enrich BM, the standard bactericide used as a positive control, showed the highest antibacterial activity against* X. axonopodis *pv.* manihotis* producing mean inhibition zones of 35.2 and 37.0 mm after 24 and 48 hours, respectively (Figure 2). Generally,* P. savastanoi* pv.* phaseolicola* and* X. axonopodis *pv.* manihotis* showed the highest and lowest susceptibility to the essential oils, respectively.

### 3.3. Antibacterial Activity of Different Concentrations of* Tagetes minuta* Essential Oils

There was a concentration-dependent inhibitory activity of EOs against the test bacteria. Thus, as the concentration of the essential oils increased, the activity of the oils against the test bacteria increased (Table 2). However, the inhibition zones produced at different concentrations of the essential oils varied from one bacterial species to another. Dose-dependent response of bacteria to essential oils of* T. minuta* has been reported in [31, 32]. Other biological activities of* T. minuta* such as tick repellency [27, 34], antioxidative and anti-inflammatory effects [30], aphidicidial activity [35], and allelopathic effects [36] have all been shown to occur in a concentration-dependent manner.

### 3.4. Dose-Response Effects of the Essential Oils on the Growth of Bacteria

**Figure 3:**
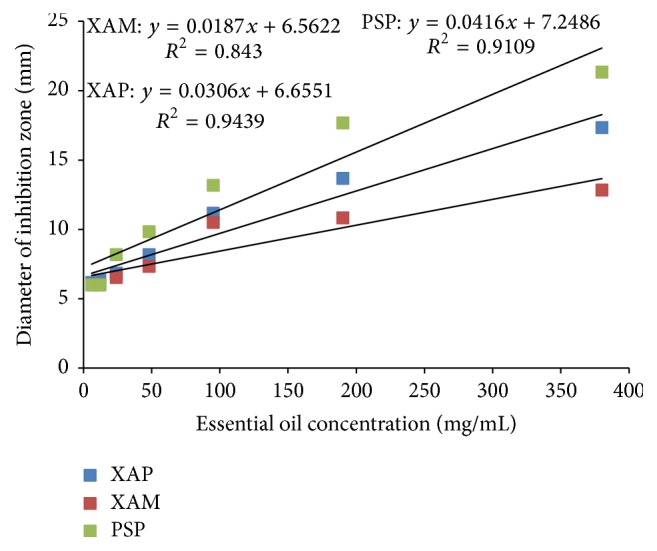
A dose-response curve of inhibition zone diameters (mm) against concentration of* T. minuta* essential oils (mg/mL) for the test bacteria. XAP:* Xanthomonas axonopodis* pv*. phaseolicola*; XAM:* Xanthomonas axonopodis* pv*. manihotis*; and PSP:* Pseudomonas savastanoi* pv*. phaseolicola.*

The dose-response model showed a significant correlation (*p* ≤ 0.05) between the concentrations of* T. minuta* essential oils and the mean inhibition zones for the three test bacteria (Figure 3). The correlation coefficient values were as follows: *R* ^2^, 0.94, *p* ≤ 0.001; *R* ^2^, 0.91, *p* ≤ 0.001; and *R* ^2^, 0.84, *p* = 0.004 for* X. axonopodis* pv.* phaseoli*,* P. savastanoi* pv.* phaseolicola*, and* X. axonopodis *pv.* manihotis*, respectively. The results thus indicated that 94, 91, and 84% of the variation in diameters of the inhibition zones in* X. axonopodis* pv.* phaseoli*,* P. savastanoi* pv.* phaseolicola*, and* X. axonopodis *pv.* manihotis*, respectively, were explained by variation in the concentration of the essential oils. It has previously been reported that antibacterial activity and indeed most biological activities of essential oils are dependent on their concentrations [31, 32]. The concentration-dependent antibacterial activity of EOs observed was attributed to changes in concentration of the active principles present in the EOs. This view is supported by several studies that have reported dose-dependent biological activities of various pure compounds isolated from essential oils [37, 38]. Thus, the concentration of the EOs increases, so does the active component present in the oils and hence there is an increase in the activity of the oils in this case represented by larger inhibition zones.

### 3.5. Minimum Inhibitory Concentrations and Minimum Bactericidal Concentrations

Minimum inhibitory concentrations (MICs) and minimum bactericidal concentrations (MBCs) of* T. minuta* essential oils on the three test bacteria are shown in Table 3. Minimum inhibitory concentrations values for the test bacteria ranged from 24 to 48 mg/mL with* X. axonopodis* pv.* phaseoli* and* P. savastanoi* pv.* phaseolicola* having the lowest value (24 mg/mL) and* X. axonopodis *pv.* manihotis* having the highest value (48 mg/mL). The estimated MICs values obtained using disc diffusion methods were 12, 24, and 48 mg/mL for* P. savastanoi* pv.* phaseolicola*,* Xanthomonas axonopodis* pv.* phaseoli*, and* X. axonopodis *pv.* manihotis*, respectively. Minimum bactericidal concentrations of* T. minuta* essential oils on the three test bacteria were in the range of 95 to 190 mg/mL.* Xanthomonas axonopodis* pv.* phaseoli* and* P. savastanoi* pv.* phaseolicola* had the lowest MBCs (95 mg/mL) while* X. axonopodis *pv.* manihotis* had the highest (190 mg/mL). The variation in the MIC values obtained using disc diffusion and tube dilution methods is indicative of the difficulty that exists when comparing antimicrobial susceptibility results, which have been obtained using different methodologies, especially in regard to the minimal inhibitory concentrations [39, 40]. In this study, differences in the solubility, diffusion, and evaporation rates of the essential oils in agar and broth media could be some of the factors that contributed to the observed differences between the two methods.

### 3.6. Chemical Composition of the Essential Oils of* Tagetes minuta*

**Figure 4:**
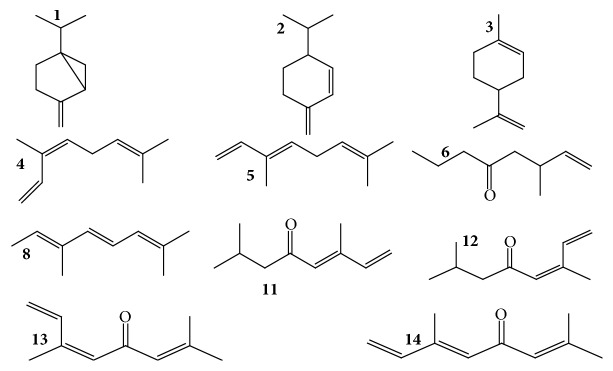
Chemical structures of monoterpenes identified in* Tagetes minuta* essential oils. (**1**) Sabinene, (**2**) *α*-phellandrene, (**3**) limonene, (**4**) (Z)-*β*-ocimene, (**5**) (E)-*β*-ocimene, (**6**) dihydrotagetone, (**8**) alloocimene, (**11**) (Z)-tagetone, (**12**) (E)-tagetone, (**13**) (Z)-ocimenone, and (**14**) (E)-ocimenone.

**Figure 5:**
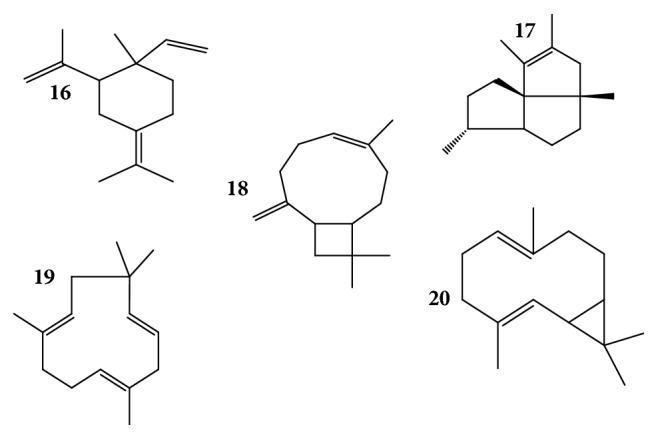
Chemical structures of sesquiterpenes identified in* Tagetes minuta* essential oils. (**16**) Elixene, (**17**) silphiperfol-6-ene, (**18**) (E)-caryophyllene, (**19**) *α*-humulene, and (**20**) bicyclogermacrene.

Twenty compounds corresponding to 96% of the total oil of* T. minuta* were identified (Figures 4 and 5). The essential oils comprised hydrocarbons mainly terpenes, a mixture of monoterpenes (70%) and sesquiterpenes (30%). The most abundant compounds identified in the essential oils were (E)-tagetone, dihydrotagetone, and alloocimene while the least abundant compound was silphiperfol-6-ene. In summary, monoterpene hydrocarbons (30%), oxygenated monoterpenes (25%), and 3 unknown monoterpenes (15%) occurred in relatively high concentrations compared to sesquiterpene hydrocarbons (25%) and nonoxygenated sesquiterpenes and one unknown sesquiterpene (1%).

Predominance of monoterpenes and sesquiterpenes in EOs of* T. minuta* in the current study was in agreement with earlier studies. A study on* T. minuta* EOs from three regions in Kenya, namely, Kasarani (Nairobi County), Bungoma (Bungoma County), and Bondo (Siaya County), found monoterpenes and sesquiterpenes hydrocarbons in the ratios of 3 : 7, 2 : 8, and 4 : 6, respectively [27]. These findings were also comparable with a number of earlier qualitative studies on the chemical composition of EOs of* T. minuta* that have identified dihydrotagetone, tagetones, ocimenes, limonene, and ocimenones as the most abundant constituents of* T. minuta* essential oils. In one such study [41], four monoterpene constituents—limonene, *β*-ocimene, dihydrotagetone, and tagetone—were found to represent more than 70% of* T. minuta* essential oil. Dihydrotagetone has been cited as one of the most abundant constituents of* T. minuta* oils from plants sampled from a wide range of countries such as Kenya [28, 42], Iran [32], Zambia [43], and the UK [44] constituting 16.7, 33.9, 30.0, and 34.3% of the total oil, respectively.

## 4. Conclusion

Development of natural antimicrobial agents is a major step towards reduction of the negative effects associated with synthetic chemical pesticides such as slow biodegradation, toxic residues in agricultural products, resistance development in targeted microorganisms, and general harmful effects on environment, humans, and animal. The objective of this study was to investigate the antibacterial activity of essential oils isolated from* T. minuta* against three plant pathogenic bacteria.* In vitro* studies revealed that the essential oils had a remarkable antibacterial activity against all the test bacteria, namely,* Pseudomonas savastanoi* pv.* phaseolicola, Xanthomonas axonopodis* pv.* phaseoli*, and* Xanthomonas axonopodis *pv.* manihotis*. This study therefore confirms the biopesticidal nature of* T. minuta* essential oils and their potential uses as cheap, safe, and effective alternative to chemical bactericides. To achieve this goal, however, the potential use of the EOs in management of bacteria plant diseases should be validated under field conditions.

## Figures and Tables

**Table 1:** Inhibition zones (mm) of *Tagetes minuta* essential oils and Enrich BM on three plant pathogenic bacteria after 24 and 48 hours.

Bacteria species	24 hours			48 hours		
	Essential oil	Enrich BM (+ve control)	DMSO (−ve control)	Essential oil	Enrich BM (+control)	DMSO (−ve control)
XAP	26.83 ± 0.60^f^	29.50 ± 0.29^e^	0.00	27.67 ± 0.88^ef^	29.50 ± 0.29^e^	0.00
XAM	26.83 ± 0.17^f^	35.17 ± 0.73^cd^	0.00	23.00 ± 1.00^g^	37.00 ± 0.58^c^	0.00
PSP	41.83 ± 0.93^b^	33.17 ± 0.93^d^	0.00	44.83 ± 0.83^a^	33.17 ± 0.73^d^	0.00

Mean	31.83	32.61		31.83	33.22	

Values are mean ± standard error of the mean for bioassays conducted in triplicate. Means followed by the same letter(s) for the two incubation regimes are not significantly different (multivariate analysis, Fisher's Protected LSD at *p* ≤ 0.05).

XAP: *Xanthomonas axonopodis *pv.* Phaseolicola*; XAM:* Xanthomonas axonopodis *pv.* Manihotis*; and PSP: *Pseudomonas savastanoi *pv.* Phaseolicola.*

**Table 2:** Inhibition zones (mm) of three bacterialspecies at different concentrations of *Tagetes minuta* essential oils after 48 hours of incubation.

Bacteria	Essential oil concentration (mg/mL) *∗* 10^2^							DMSO (−ve control)	MIC (mg/mL)
	3.8	1.9	0.95	0.48	0.24	0.12	0.06		
XAP	17.33 ± 0.60	13.67 ± 1.01	11.17 ± 0.60	8.17 ± 0.17	≤8.00	≤8.00	≤8.00	0.00	24.00
XAM	12.83 ± 0.60	10.83 ± 0.93	10.50 ± 0.29	≤8.00	≤8.00	≤8.00	≤8.00	0.00	48.00
PSP	21.33 ± 0.44	17.67 ± 0.17	13.17 ± 0.60	9.83 ± 0.16	8.17 ± 0.17	≤8.00	≤8.00	0.00	12.00

Values are means ± standard error of the mean for bioassay conducted in triplicate.

XAP: *Xanthomonas axonopodis *pv.* phaseolicola*; XAM: *Xanthomonas axonopodis *pv.* manihotis*; PSP: *Pseudomonas savastanoi *pv.* phaseolicola*; and MIC: minimum inhibitory concentration.

**Table 3:** Minimum inhibitory concentrations and minimum bactericidal concentrations of essential oils of *Tagetes minuta* on three plant pathogenic bacteria.

Tube/plate number	EOs concentration (mg/mL) *∗* 10^2^	Growth of bacteria in MHB tubes			Growth of bacteria in MHA plates		
		XAP	XAM	PSP	XAP	XAM	PSP
#1	Crude EOs	−	−	−	−	−	−
#2	3.8	−	−	−	−	−	−
#3	1.9	−	−	−	−	−	−
#4	0.95	−	−	−	−	+	−
#5	0.48	−	−	−	+	+	+
#6	0.24	−	+	−	+	+	+
#7	0.12	+	+	+	+	+	+
#8	0.06	+	+	+	+	+	+
#9	0.03	+	+	+	+	+	+
#10	0.015	+	+	+	+	+	+
#11	−ve control	+	+	+	+	+	+
#13	+ve control	−	−	−	−	−	−

[+]: growth; [−]: no growth of the bacteria; and XAP: *X. axonopodis *pv.* phaseoli*, XAM: *X. axonopodis *pv. *manihotis,* and PSP: *P. savastanoi *pv*. Phaseolicola*.

MHB: Muller-Hinton broth; MHA: Muller-Hinton Agar; tube 1: bacterial inoculum and undiluted crude essential oils; tubes 2 to 10: MHB, bacterial inocula, and essential oils of different concentrations; tube 11: bacterial inoculum and MHB (−ve control); and tube 13: bacterial inoculum, MHB, and the standard bactericide (+ve control).
